# NiS ultrafine nanorod with translational and rotational symmetry

**DOI:** 10.1093/nsr/nwae175

**Published:** 2024-05-16

**Authors:** Jianxin Kang, Qi Hu, Ruixuan Zhang, Ang Gao, Zhongning Huang, Ziming Su, Ke Pei, Qinghua Zhang, Li-Min Liu, Renchao Che, Lin Gu, Er-Jia Guo, Lin Guo

**Affiliations:** School of Chemistry, Beihang University, Beijing 100191, China; School of Chemistry, Beihang University, Beijing 100191, China; Laboratory of Advanced Materials, Shanghai Key Lab of Molecular Catalysis and Innovative Materials, Academy for Engineering & Technology, Fudan University, Shanghai 200438, China; Zhejiang Laboratory, Hangzhou 311500, China; Beijing National Center for Electron Microscopy and Laboratory of Advanced Materials, School of Materials Science and Engineering, Tsinghua University, Beijing 100084, China; School of Chemistry, Beihang University, Beijing 100191, China; School of Chemistry, Beihang University, Beijing 100191, China; Laboratory of Advanced Materials, Shanghai Key Lab of Molecular Catalysis and Innovative Materials, Academy for Engineering & Technology, Fudan University, Shanghai 200438, China; Beijing National Laboratory for Condensed Matter Physics and Institute of Physics, Chinese Academy of Sciences, Beijing 100190, China; School of Physics, Beihang University, Beijing 100191, China; Laboratory of Advanced Materials, Shanghai Key Lab of Molecular Catalysis and Innovative Materials, Academy for Engineering & Technology, Fudan University, Shanghai 200438, China; Zhejiang Laboratory, Hangzhou 311500, China; Beijing National Center for Electron Microscopy and Laboratory of Advanced Materials, School of Materials Science and Engineering, Tsinghua University, Beijing 100084, China; Beijing National Laboratory for Condensed Matter Physics and Institute of Physics, Chinese Academy of Sciences, Beijing 100190, China; School of Chemistry, Beihang University, Beijing 100191, China

**Keywords:** separated symmetry, NiS, anisotropy structure, atomic machining, magnetic domain

## Abstract

Anisotropy is a significant and prevalent characteristic of materials, conferring orientation-dependent properties, meaning that the creation of original symmetry enables key functionality that is not found in nature. Even with the advancements in atomic machining, synthesis of separated symmetry in different directions within a single structure remains an extraordinary challenge. Here, we successfully fabricate NiS ultrafine nanorods with separated symmetry along two directions. The atomic structure of the nanorod exhibits rotational symmetry in the radial direction, while its axial direction is characterized by divergent translational symmetry, surpassing the conventional crystalline structures known to date. It does not fit the traditional description of the space group and the point group in three dimensions, so we define it as a new structure in which translational symmetry and rotational symmetry are separated. Further corroborating the atomic symmetric separation in the electronic structure, we observed the combination of stripe and vortex magnetic domains in a single nanorod with different directions, in accordance with the atomic structure. The manipulation of nanostructure at the atomic level introduces a novel approach to regulate new properties finely, leading to the proposal of new nanotechnology mechanisms.

## Teaser

A new type of crystal structure is reported, which exhibits rotational symmetry in the radial direction and translational symmetry in the axial direction, surpassing the conventional crystalline structures.

## INTRODUCTION

Symmetry is a fundamental concept within materials science and solid-state physics, assuming a vital role in establishing structure-property correlations [1–4]. In relation to symmetry, the ordered structures can be examined through close sphere packings with partially filled voids. Alternatively, they can be analyzed using packings of polyhedra, considering the connectedness of structures and substructures within frameworks of varying dimensions. A crystal is a solid with atoms situated in a repeating or periodic array over large atomic distances with long-range order, in which the atoms will position themselves in a repetitive three-dimensional pattern [5]. Thus, it is a system with translational and rotational symmetry, in which physical observables, such as the energy spectrum and the locations of atoms, are periodic under the action of discrete translations and rotations [6,7]. Furthermore, lattice complexities, graphs, tilings, and topological properties can be leveraged to investigate these structures [8–13]. Distinct from crystals, quasicrystals possess rotation symmetry but lack lattice periodicity, without translational symmetry [14,15]. Nonetheless, captivating physical phenomena often arise when a specific form of symmetry in the system is disrupted, either through spontaneous processes or external influences. For example, broken, partial, or incomplete symmetries result in such a diverse range of states/phenomena as Higgs bosons, superconductivity, ferromagnetism, and magnetoresistance due to weak localization [16–20].

Although the exploration and design of new symmetries are crucial for unlocking new functionalities in materials, there are currently limited reports on novel symmetrical materials. The regulation of the atomic configuration of nanomaterials mainly focuses on the geometrical morphology design or chemical component adjustment [21–24]. The difficulty in conducting each atom's location for unusual symmetries makes establishing structure-relevant engineering strategies and mechanisms unclear. Even though some pioneering achievements have been made in the atomic symmetrical design, the lattice structure should consistently possess translational symmetry and/or rotational symmetry in any direction [25,26]. The synthesis of a novel atomic arrangement featuring distinct symmetries in two directions has yet to be achieved. Exploring the relationship between this newfound symmetry and spin textures could serve as a valuable complement to the current crystallographic principles.

Here, we report the successful synthesis of an ultrafine nanorod made of NiS that exhibits a unique separation of symmetry in both the axial and radial directions. The rotational symmetry of the atomic arrangement in the radial direction and the divergent translational symmetry in the axial direction push the nanorod beyond the confines of well-established crystalline structures. To the best of our knowledge, this is the first exhibition of a complex atomic configuration to realize direction-dependent separated symmetry in a single structure. Due to its unique structure, the long-range ordered atoms along the nanorod's longitudinal axis result in a preferred orientation of spins and aligned magnetic moments in the same direction. Conversely, the circular arrangement impedes the alignment consistency of spins and magnetic moments, leading to their arrangement forming a closed loop in the radial direction. These findings offer fresh insights into the programming of atomic location, thereby facilitating the exploration of diverse application potentials for low-dimensional nanomaterials.

## RESULTS AND DISCUSSION

**Figure 1.**
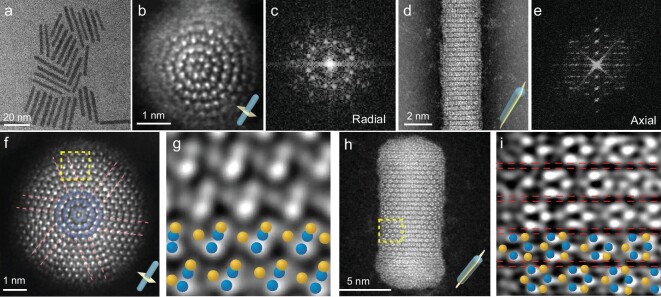
Morphologies of NiS ultrafine nanorods. (a) TEM image of the ultra-fine NiS nanorods with diameters of ∼2.7 nm. (b) ADF-STEM image and (c) corresponding FFT pattern of the ultra-fine nanorod in the radial direction. (d) ADF-STEM image and (e) corresponding FFT pattern of the ultra-fine nanorod in the axial section. (f–g) ADF-STEM image and the enlarged image of a thicker NiS nanorod with a diameter of ∼6 nm in the radial direction. (h–i) ADF-STEM image and the enlarged image of a thick NiS nanorod in the axial direction. The blue and yellow spheres in (g) and (i) represent the Ni and S atoms, respectively.

Symmetry is the key factor in the long history of recognition of materials, from crystal to amorphous materials and quasi-crystal. However, the synthesis of materials with distinct symmetries in different directions within a single structure remains unexplored. To achieve this objective, ultrafine NiS nanorods with a diameter of ∼2.7 nm and a length of ∼30 nm (Fig. 1a) were prepared (see Methods). Based on the atomic-resolved annular dark-field scanning transmission electron microscopy (ADF-STEM) image, the radial section (Fig. 1b) of the nanorod clearly reveals five circles of regular atomic rings with a spacing of ∼0.28 nm (Fig. S1). The structure of the NiS nanorod deviates from conventional periodic crystal lattices [27], which carries the rotational symmetry in the radial direction but loses the translational symmetry. In contrast to the traditional 5-fold twinned crystal or typical 5-fold symmetry of a quasi-crystal, the radial arrangement of the atoms has a 6-fold symmetry [28,29]. Moreover, the fast Fourier transformation (FFT) pattern of the nanorod reveals a distinctive atomic arrangement, as evidenced by the presence of 12 non-isometric diffraction spots organized into six groups within the first circle (Fig. 1c). This pattern deviates from the typical rhombic matrix, providing further confirmation of the unconventional atomic arrangement. Conversely, when observed from a side view, the NiS nanorod exhibits a regular translational periodicity (Fig. 1d). The lamellar structure with an interlayer spacing of 4.8 Å is evident in Fig. S2. The typical translational periodicity in the axial direction is a prerequisite for the clear imaging in the radial direction, which provides periodic repeating atoms as columns. However, the absence of translation periodicity in the radial direction causes indistinguishable atomic imaging in the axial direction. The FFT pattern demonstrates typical periodic spots along the axial direction, corresponding to the existence of translational symmetry (Fig. 1e). In contrast, the horizontal streaks indicate that the projected periodicity of the atoms in the radial direction is disordered, with broken symmetry in the radial direction. As a result, this nanorod realizes separated rotational symmetry in the radial direction and translational symmetry in the axial direction, which further extends beyond the conventional classification of crystalline structures.

In order to explore the growth mechanism of the NiS nanorod, the precursors of different stages before the nanorods’ formation were characterized. It could be found that when the temperature was raised to 160°C, the product was disjunctive amorphous clusters with diameters of ∼1 nm (Fig. S3). They were supposed to be amorphous NiS precursors, while Ni was from reactant Ni(acac)_2_ and S was from the decomposition of 1-dodecanethiol. The high specific surface area and highly unsaturated coordination structure of the amorphous clusters resulted in the linking of 1-dodecanethiol on the surfaces, showing as separated small particles in the ADF-STEM image. Thus, the formation of the NiS nanorod is supposed to be the assembly of the initial clusters restricted by 1-dodecanethiol. The strong wrapping effect of 1-dodecanethiol on the surface of NiS nanorods also enabled the outermost layer of the NiS nanorod to maintain its amorphous disordered structure (Fig. 1b). Due to the ultrafine diameter of NiS nanorods and the spatial confinement effect of 1-dodecanethiol chains wrapped around the surface, the atoms in the radial direction of the nanorods show a spatially optimized radial arrangement instead of the conventional energy-optimized three-dimensional lattice arrangement.

Although the electron energy loss spectroscopy mapping (Fig. S4) and inductively coupled plasma-atomic emission spectrometer results indicate a homogeneous distribution of Ni and S elements with an atomic ratio of 1:1, the distinctive radial atomic arrangement of the ultrathin nanorods, lacking a basic unit cell structure, poses challenges for the analysis of its atomic structure. To address this challenge, the growth time was extended, successfully preparing a mature nanorod with a thicker diameter of ∼6 nm. The new structure maintains the ring geometry in the innermost three layers. Apart from it, the exterior of the nanorod has evolved to a 6-fold twinned structure as indicated by twin boundaries (Fig. 1f). Each twin region is highly ordered with a translational symmetry (Fig. 1g), indicating the formation of a unique crystal structure. The FFT pattern of the cross-section figure also shows sharp 6-fold diffraction spots, indicating better crystallinity (Fig. S5a). If inner layers were framed, a similar FFT pattern to the one shown in Fig. 1c was displayed, revealing similar ring geometry in the innermost layers (Fig. S5b). Due to the translational order in the external part, the edge of the thicker rod could be atomically recognized from the side view (Fig. 1h), different from the ultrafine nanorod. Highly ordered hexagonally arranged atoms with good translational symmetry can also be identified in an enlarged image of Fig. 1i. Thus, an interesting graphic was displayed with indistinguishable atomic imaging in the core and clearly atomic imaging in the shell. Nevertheless, it also showed a regular lamellar structure with interlayer spacing of 4.6 Å (Fig. S6).

To analyze the atomic structure, X-ray diffraction (XRD) was employed for the as-prepared NiS thicker nanorod. While the XRD patterns of the material under investigation did not match any known standard materials, they displayed a resemblance to those of β-NiS (rhombohedral, *R*3*m*), which exhibits a square-pyramid structure [30,31] (Fig. S7). Furthermore, the distinct hexagonal pattern consisting of three Ni atoms (bright spots, covered with blue spheres) and three S atoms (gray spots, covered with yellow spheres) observed in Fig. 1i displayed a notable resemblance to the atomic arrangement of the pentacoordinate β-NiS along the [100] projection. Additionally, combining the TEM images with the DFT computational model shows that the axial direction of the nanorods is along the [010] direction, which is the direction of maximum growth (Fig. S8). This observation offers a valuable clue for analyzing the material's structure.

**Figure 2.**
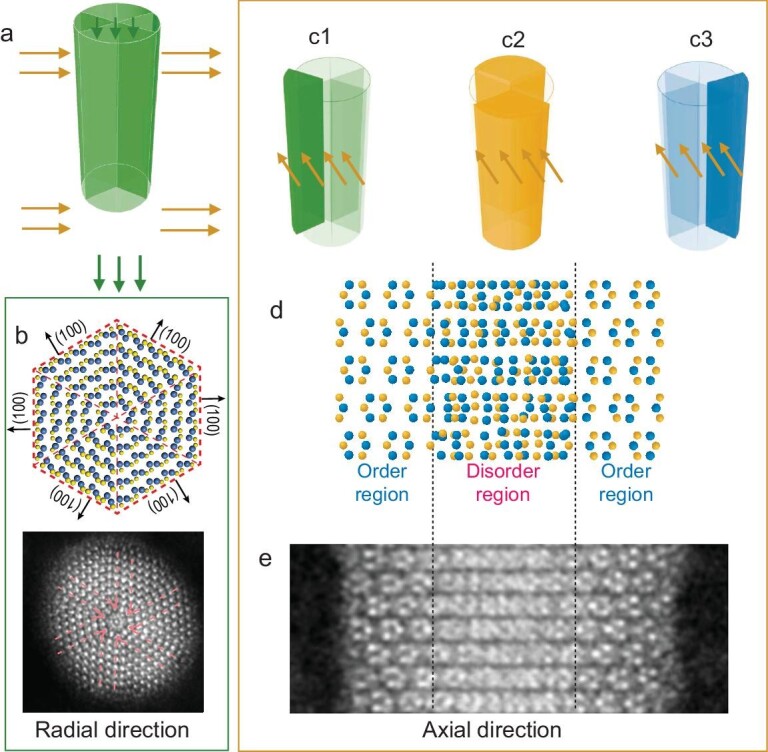
Constructed atomic configuration of the NiS nanorod. (a) The schematic model of the NiS nanorod with six regions. (b) Top view of the NiS ultrafine nanorod (upper panel), compared with the corresponding ADF-STEM image (lower panel). The optimized structure is composed of six similar triangular rods, each exposing a (100) surface. The two of six triangular rods in the right and left sides are set to align with the axial. The number of circles is dependent on the number of layers, and the five-layer structure was employed in the simulation. (c–e) The side view is shown by three groups to better understand the internal structure with a schematic model. The rods in the two sides are in (c1) and (c3), which mainly correspond to the order region in (d) and (e). The other four regions are in (c2), and projection of the nanorod NiS is the disorder region in (d) and (e). The overlapped and unoverlapped regions are projected by the triangular regions in the center and on the two sides, respectively. The blue and yellow spheres represent the Ni and S atoms, respectively.

To better comprehend the intricate atomic structure, first-principles calculations were carried out to examine the surface energies of all low-index surfaces of β-NiS (Fig. S9). The computed surface energies of β-NiS suggest that the (100) surface possesses the lowest value due to the significant reconstructions in the surface (Fig. S10). As a result, β-NiS tends to maximize the presence of the (100) surface during its growth. Based on the experimental results and Wulff theorem, the atomic model of the NiS nanorod was constructed with six similar triangle rods, exposing the (100) surfaces. As illustrated in Fig. 2 and Fig. S11, the initial model of the proposed atomic structure was automatically optimized to the final structure, with an obvious reconstruction. As shown in the top view, the original structure turns into a characteristic of circles from inner to outer. The circles are composed of Ni atoms, which corresponds to the bright spots in the ADF-STEM images in Fig. 2b. Meanwhile the spacing between the circles is 2.8 Å, which is in good agreement with the experimental result (Fig. S12). Additionally, the number of circles depends on the number of layers of NiS. Simulation results for NiS nanorods of varying diameters reveal that thinner nanorods tend to display a more pronounced circle-like structure (Fig. S13), which is also in agreement with the ADF-STEM images (Fig. 1b and f).

The side view of the NiS ultrafine nanorod shows a stripe feature along the (010) direction, with the stripe spacing of 4.8 Å (Fig. S12), which is consistent with the experiment findings. Moreover, the edge and central regions of the nanorod exhibits distinct projection features (Fig. 2c). For the edge regions, the two opposite triangular rods of the 6-fold symmetry nanorod are prominently projected on the borders aligning with the axial (Fig. 2c1 and c3), and exhibit a pattern of 3Ni-3S six-membered rings (Fig. 2d). The feature observed in the edge regions is consistent with the experimental image of the six atomic rings depicted in Fig. 1i: three atoms of the ring correspond to Ni (light spots), while the remaining three atoms of the ring represent S (dark spots) (Fig. 2e). However, in the central region of the nanorod, the transmission image exhibits a predominantly disordered distribution because the remaining four of six polycrystalline regions are not aligned with the axial, thus the projection of the four regions does not show ordered structure (Fig. 2c and d).

To confirm the geometric structure, X-ray absorption fine structure (XAFS) spectroscopy is employed. The white line peak in the Ni *K*-edge X-ray absorption near-edge structure (XANES) spectrum is attributed to the electronic transition from the 1*s* orbital to the unoccupied 4*p* orbitals, and the oxidation state of Ni is determined by the energy position of the white line peak [32,33]. The energy position of the as-prepared NiS nanorod demonstrated lower energy in comparison to standard NiS, indicating a lower oxidation state than Ni^2+^ (Fig. S14a). It suggested a unique structure of the as-prepared NiS nanorod compared to the conventional classification of crystalline structures. The Fourier transformed extended X-ray absorption fine structure (FT-EXAFS) spectra displayed a single peak in the as-prepared NiS nanorod, differing from that of the NiS reference (Fig. S14b). The absence of Ni-Ni scattering peaks suggested an elongation of the distance between Ni atoms and their adjacent counterparts, representing a reduced crystalline symmetry [34,35]. Meanwhile, the shifted Ni-S peak also reveals an enlarged atomic distance compared to the standard NiS (Table S1), displaying an increased distortion. These distinctive pentacoordinate atomic structures offer an additional avenue for regulating magnetism.

**Figure 3.**
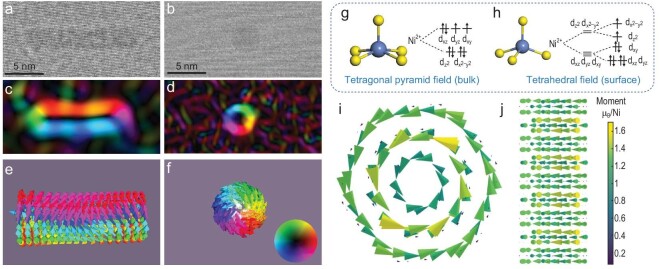
Stripe-vortex coupled magnetic order of the NiS nanorod. TEM electron holograms and reconstructed distribution of magnetic domains of NiS nanorod along the axial (a and c) and the radial (b and d) directions, respectively. Simulated distribution of magnetic moments in axial (e) and radial (f) directions, respectively. (g and h) The Ni-S crystal field in crystal β-NiS and on the reconstructed (100) surface. (i and j) The calculated noncollinear magnetism for the NiS nanorod with the three-layer structure, shown along the radial and axial directions, respectively.

The decoupling of symmetry in radial and axial directions would induce strong magnetic anisotropy and construct the unique magnetic structure of the ultrafine nanorod. The unprecedented ferromagnetic order and multidomain structure in the as-prepared ultrafine NiS nanorod is successfully realized via subtly-managed atomic arrangement and anisotropic symmetry, as proven by the TEM electron hologram and micromagnetic simulation (Fig. 3, Fig. S15). Figure 3a and c show the distribution of magnetic domains along the axial direction where color and brightness present the direction and the strength of magnetization perpendicular to the electron beam, respectively. Blue and red bands on either side of a NiS nanorod indicate the existence of two antiparallel stripe domains. As for the radial direction (Fig. 3b and d), the reconstructed hologram shows an obvious color ring, corresponding to a vortex domain. Furthermore, the micromagnetic simulation demonstrates the formation of both the stripe and vortex domains in two directions, which is well consistent with the experimental result. These unique magnetic structures prove that the spin alignment follows the intrinsic atomic arrangement. Along the long axis, long-range ordered atoms produce the preferred orientation of spins and aligned magnetic moments in the same direction (Fig. 3e), accompanied by formation of the domain wall. In the radial direction, the circular arrangement restrains the orientation consistency of spins and the magnetic moments are thereby arranged as a closed loop (Fig. 3f). Hence, the symmetry separation observed in the NiS ultrafine nanorod showcases the integration of multiple magnetic orders, a phenomenon previously unobserved in conventional crystals, quasicrystal, and amorphous materials.

As discussed above, the NiS nanorod undergoes surface reconstruction during formation. Within the crystal β-NiS, Ni^2+^ exhibits a square-based pyramid crystal field with five coordinated sulfur atoms (Fig. 3g), with t_2 g_ orbitals fully occupied and e_g_ orbitals half occupied. Conversely, on the (100) surface, the Ni-S crystal field transforms into a tetrahedral field with only four coordinated sulfur atoms (Fig. 3h), and the surface Ni^2+^ displays a unique electronic structure with a fully occupied e_g_ orbital and partially filled t_2 g_ orbital. Interestingly, the atomic magnetic moments for bulk Ni^2+^ in the square-pyramidal field (1.31 μB) was increased to 1.55 μB for surface Ni^2+^ in the tetrahedral field. Thus, the symmetry separated NiS ultrafine nanorod could demonstrate novel magnetic properties different from bulk samples. To further investigate the effects of the magnetic properties induced by the symmetry separated structure, both collinear and noncollinear magnetic configurations were considered, and the Stripe-Vortex mode exhibits the lowest energy (Fig. 3i and j and Fig. S16), consistent with our TEM electron hologram. Meanwhile the exterior Ni atoms demonstrate the obvious large magnetic moments due to surface reconstruction.

The mechanisms behind symmetry-determined unique magnetic structures can be attributed to the following two aspects. First, the special in-plane atomic arrangement can be analogized to a series of concentric circles. The distance between atoms on adjacent circles is larger than that on the same circle, which leads to a decreased exchange energy. Second, the typical rotational symmetry induces the isotropic magnetizability, while the translational symmetry produces the anisotropic one. Due to spin-orbital coupling, the orbital motion is strongly coupled to the lattice degree of freedom, allowing the lattice configuration and symmetry to influence the spin direction of electrons indirectly. As a result, symmetry plays a crucial role in determining the preferred direction of magnetization, thereby affecting the magneto-crystal anisotropy energy. Consequently, along the axial direction, the presence of strong exchange energy and anisotropic magnetizability jointly promotes the formation of stripe domains. Conversely, the relatively weaker exchange interaction combined with isotropic magnetizability leads to the generation of vortex domains in the radial direction.

**Figure 4.**
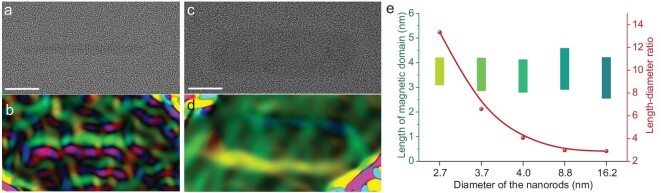
The magnetic domain structure of the NiS nanorod. A thinner nanorod with a diameter of 2.7 nm (a and b) and a thicker NiS nanorod with a diameter of 16.2 nm (c and d) are preformed to display the unified ultra-small critical size of the magnetic domains. Scale bars in (a) and (c) are 1 nm. (e) The statistic size of the magnetic domains (left y-axis) and the length-diameter ratios (right y-axis) of the NiS nanorods with different diameters.

This unique phenomenon which combines the stripe and vortex magnetic domains in a single structure is further confirmed to be universal in nanorods with different diameters. In the range of diameters from 2.7 nm to 16.2 nm, NiS nanorods showcase a consistent distribution of magnetic domains along the axial direction. Specifically, two antiparallel stripe domains are observed on either side of the nanorod, maintaining their separate orientations (Fig. 4a–d). These results indicate that the symmetric separation significantly influences the anisotropic magnetism order and magnetic domain structure of the ultrafine nanomaterials. Our method and the proposed mechanism pave the way for the fabrication of ultra-small magnetic domains, opening up new possibilities for investigating magnetism at the frontier of research. Anyway, the symmetric anisotropy can break through the bottleneck of the finite-size effect described in conventional condensed matter physics. Conventional magnets in a recording medium hold a critical size below which their magnetism deteriorates dramatically and inhibits storing digital information in memories. By employing our approach of symmetry separation, the critical size can be significantly reduced, such as achieving a diameter smaller than 3 nm for NiS (Fig. 4e). In comparison, the minimum size for pure Ni is 14.6 nm, and for nickel sulfide it is 55.7 nm [36,37]. Both of which were much larger.

The size of magnetic domains in nanostructures typically does not change significantly with diameter due to the dominance of other factors such as surface energy minimization and magnetic anisotropy. In nanostructures, the surface energy plays a crucial role in determining the size of magnetic domains. As the diameter decreases, the surface-to-volume ratio increases, leading to a stronger influence of surface energy on domain formation. This can result in the stabilization of domain size despite changes in the diameters of nanorods. Additionally, magnetic anisotropy, which is the tendency of a material to preferentially align its magnetic moments in a particular direction, can also influence domain size. The magnetic anisotropy energy becomes more dominant as the size of the nanorod decreases, helping to maintain relatively constant domain sizes. Overall, while changes in the diameter of nanorods can affect some properties of magnetic domains, other factors such as surface energy minimization and magnetic anisotropy play more significant roles in determining domain size in these nanorods.

At the same time, the aforementioned magnetic order mechanism, which is dependent on symmetry, provides valuable guidance for an innovative approach to engineer the intrinsic magnetic properties of materials. In contrast, the traditional strategy of morphological regulation primarily focuses on surface protuberances and edges for designing magnetic domain structures. This approach takes advantage of the large demagnetization energy present in these regions, promoting the formation of complex local domain structures. Our symmetric operation can manage the intrinsic magnetic configuration based on atomic arrangement without altering the morphology and constituent, which provides a new path to discover new magnetic physics mechanisms and promotes the development of a magnetic recording medium. In addition, the magnetic vortex domains along the radial axis intrinsically impede the perturbation of the magnetic fields. This characteristic confers advantages in terms of data stability and long-term storage. Additionally, the magnetic anisotropy enables the magnetization to be switched along the axial direction, a crucial aspect for secure data transfer and storage.

## CONCLUSION

Consequently, we have successfully synthesized a distinctive NiS nanorod with a diameter of sub-3 nm characterized by a separated atomic symmetry. The anisotropic symmetry involving both axially translational symmetry and radially rotational symmetry extends beyond the well-established crystalline structures found in nature, encompassing neither crystals nor quasicrystals. The anisotropic symmetry exhibited by NiS nanorods results in remarkably distinct stripe and vortex magnetic domains along two directions. This represents a pioneering demonstration of intricate magnetic configurations in a finely engineered nanomaterial, achieved through specialized symmetric operations. These findings illuminate new possibilities for programming nanomagnetic textures and provide valuable insights for exploring diverse application potentials of low-dimensional magnetic materials.

## Supplementary Material

nwae175_Supplemental_File
