# Supplementary material for: NiS ultrafine nanorod with translational and rotational symmetry
Source: Natl Sci Rev. 2024 May 16;11(7):nwae175. doi: 10.1093/nsr/nwae175 (PMC11173186; doi:10.1093/nsr/nwae175)
Supplement: nwae175_Supplemental_File [file nwae175_supplemental_file.pdf]

Supplementary File for

NiS ultrafine nanorod with translational and rotational symmetry

Jianxin Kang[1]†, Qi Hu[1]†, Ruixuan Zhang[2, 3]†, Ang Gao[4]†, Zhongning Huang[1]†, Ziming Su[1], Ke Pei[2], Qinghua Zhang[5], Li-Min Liu[6], Renchao Che[2, 3]*, Lin Gu[4]*, Er-Jia Guo[5]* and Lin Guo[1]*

[1]School of Chemistry, Beihang University, Beijing 100191, China.

[2]Laboratory of Advanced Materials, Shanghai Key Lab of Molecular Catalysis and Innovative Materials, Academy for Engineering & Technology, Fudan University, Shanghai 200438, China.

[3]Zhejiang Laboratory, Hangzhou 311500, China.

[4]Beijing National Center for Electron Microscopy and Laboratory of Advanced Materials, School of Materials Science and Engineering, Tsinghua University, Beijing 100084, China.

[5]Beijing National Laboratory for Condensed Matter Physics and Institute of Physics, Chinese Academy of Sciences, Beijing 100190, China.

[6]School of Physics, Beihang University, Beijing 100191, China.

*Correspondence authors. E-mails: rcche@fudan.edu.cn; lingu@tsinghua.edu.cn; ejguo@iphy.ac.cn; guolin@buaa.edu.cn

†Equally contributed to this work.

Materials and Methods

Materials

Nickel acetylacetonate ($Ni(acac)_2$), Diphenyl oxide ($C_{12}H_{10}O$) were purchased from J&K Chemicals. 1-Dodecanethiol ($C_{12}H_{26}S$) was purchased from Alfa Aesar. Ethanol(C_2H_5OH) and n-hexane(C_6H_{14}) were purchased from Beijing Chemical Works. All chemical reagents used in this experiment were analytical grade and used without further purification.

Characterization

Transmission electron microscopy (TEM) images were carried out on JEOL JEM-2100 microscopes. X-ray powder diffraction (XRD) patterns were obtained by a Shimadzu LabX-6000 X-ray diffractometer and the Data were collected in Bragg-Brentano mode with a scan rate of 1 ° min^{-1}. LAADF STEM imaging experiments were carried out using a 200 kV STEM with a sub-Å resolution (ARM200FC, JEOL) operated at 200 kV. For STEM observations, we adopted a probe size of approximately 0.78 Å, a probe convergence angle of approximately 25 mrad, and collection semi-angles for LAADF imaging of 30-120 mrad.

Synthesis of NiS Ultrafine Nanorod

In a typical synthesis, 0.0321 g (0.125 mmol) Nickel acetylacetonate was dissolved into 5 mL diphenyl oxide at room temperature in a three-neck flask. After ultrasonicated for 5 min, the flask was transferred into a water-bath at 40℃ and kept for 10 min. Then 5 mL 1-Dodecanethiol was added dropwise into the above solution and maintained heating 40℃ for 30 min. Subsequently, the flask was heated to 220℃ for 1 h under magnetic stirring. After that, the mixture was centrifugated at 13000 rpm for 5 min and washed with ethanol for the first time and n-hexane for following two times.

TEM electron holography

The TEM electron holography assists in revealing the magnetic moment orientation of our sample, based on the division of the two beams from the field emission electron gun by the interference of the prism. One beam is propagated into the vacuum, providing the information of the reference, while the other beam is transmitted through the sample. The information encoded inside the sample can be exfoliated by the extraction of the vacuum image from the sample overlapping and vacuum part to cultivate the magnetism electron holography images with the pure magnetism information in the sample based on the equation of:

$$\Phi_{mag} = \int_{obj-path} \vec{A}\, d\vec{s} - \int_{ref-path} \vec{A}\, d\vec{s} = \oint_{area} \vec{A} d\vec{s}$$

In which the Φ_{mag} indicates the magnetic flux inside the sample overlapping area with the vacuum part, surface integrated by the magnetism.

Computational method

The calculations were performed using first-principles density functional theory (DFT), as implemented in the Vienna Ab Initio Simulation Package (VASP) (1, 2). The general gradient approximation of Perdew-Burke-Ernzerhof (GGA-PBE) functional was used to describe the exchange-correlation (3). The DFT+U was employed with the value of 6.2 eV for the 3d orbitals of Ni. A plane wave cutoff of 500 eV was applied to expand the electron wave functions. The vacuum layers were set to 10 Å to avoid periodic interaction. The supercell with a = 30.00 Å, b = 9.46 Å and c = 30.00 Å was employed for NiS nanorods, and the reciprocal space was sampled using a $1 \times 2 \times 1$ mesh grid by using Monkhorst-Pack k-points scheme (4). The structures were relaxed until the variation of the total energy was smaller than 10^{-6} eV and all force on each atom was less than 0.01 eV/Å. The Becke-Johnson damping function (5, 6) was used to describe the van der Waals (vdW) interaction. For the structural optimization of NiS, linear magnetism was uniformly adopted in our calculations. Furthermore, the most stable electronic structure of the NiS nanorod configuration was determined by considering the different initial noncollinear magnetisms.

To explore the atomic structures of the large NiS nanorods for 4 or 5 layers, we employed the CP2K/Quickstep package, which is more efficient to simulate the large supercell (7). The similar parameters and criteria were employed in these two packages. The exchange correlation energy was carried out within the GGA-PBE (8). The norm-conserving Goedecker-Teter-Hutter (GTH) pseudopotentials was used to describe the core electrons (9). The correlation energy (U) for the 3d orbitals of Ni also set as 6.2 eV. The NiS nanorod was modeled using a cell with dimensions of 30.00 Å along a axis, 19.50 Å along b axis and 45.00 Å along c axis. Gaussian functions with molecularly optimized double-zeta polarized basis sets (MOLOPT-DZVP) were adopted for expanding the wave function of Ni $3p^6 4s^2 3d^8$ and S $3s^2 3p^4$ electrons (10).

Reference:

1. Kresse, G. & Furthmüller, J. Efficiency of ab-initio total energy calculations for metals and semiconductors using a plane-wave basis set. Comput. Mater. Sci. 6, 15–50 (1996).

2. Kresse, G. & Furthmüller, J. Efficient iterative schemes for ab initio total-energy calculations using a plane-wave basis set. Phys. Rev. B 54, 11169 (1996).

3. Perdew, J. P. et al. Atoms, molecules, solids, and surfaces: applications of the generalized gradient approximation for exchange and correlation. Phys. Rev. B 46, 6671 (1992).

4. Monkhorst, H. J. & Pack, J. D. Special points for Brillouin-zone integrations. Phys. Rev. B, 13, 5188-5192, (1976).

5. Dion, M., Rydberg, H., Schroder, E., Langreth, D. C. & Lundqvist, B. I. Van der Waals density functional for general geometries. Phys. Rev. Lett. 92, 246401 (2004).

6. Klimes, J., Bowler, D. R. & Michaelides, A. Van der Waals density functionals applied to solids. Phys. Rev. B 83, 195131 (2011).

7. VandeVondele, J.; Krack, M.; Mohamed, F.; Parrinello, M.; Chassaing, T.; Hutter, J. Quickstep: Fast and Accurate Density Functional Calculations Using a Mixed Gaussian and Plane Waves Approach. Comput. Phys. Commun. 2005, 167, 103-128.

8. Perdew, J. P.; Burke, K.; Ernzerhof, M. Generalized Gradient Approximation Made Simple. Phys. Rev. Lett. 1996, 77, 3865-3868.
9. Goedecker, S.; Teter, M.; Hutter, J. Separable Dual-Space Gaussian Pseudopotentials. Phys. Rev. B 1996, 54, 1703-1710.
10. VandeVondele, J.; Hutter, J. r. Gaussian Basis Sets for Accurate Calculations on Molecular Systems in Gas and Condensed Phases. J. Chem. Phys. 2007, 127, 114105.

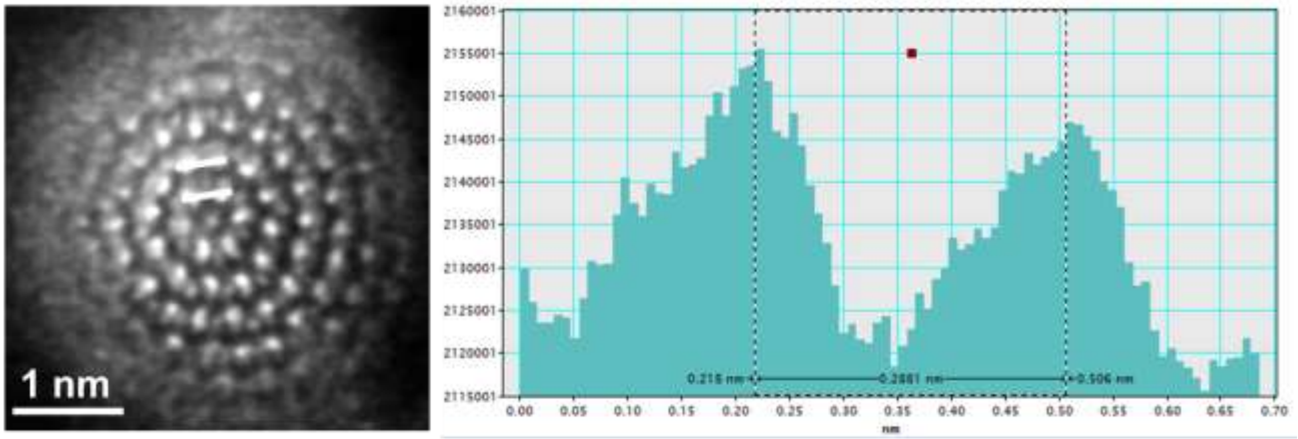

Fig. S1. The spacing between two circles is measured to be ~0.28 nm.

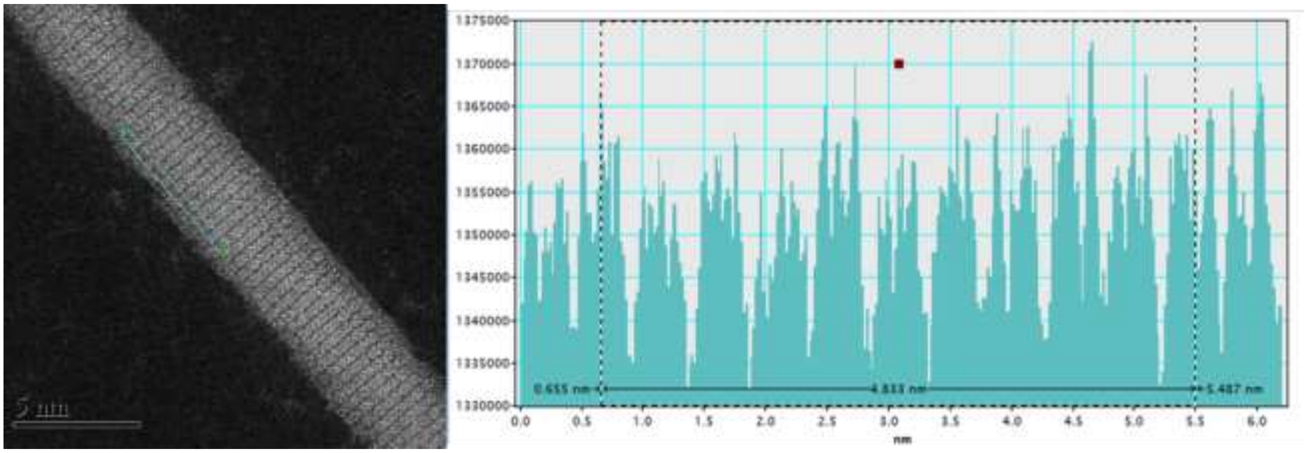

Fig. S2. The interlayer spacing of the lamellar structure form side view of NiS ultrafine nanorod is measured to be ~0.48 nm.

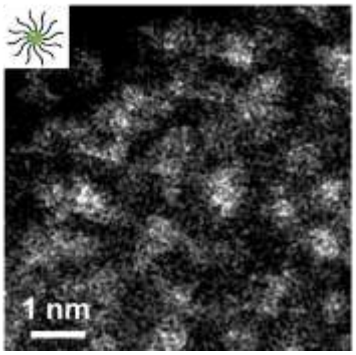

Fig. S3. ADF-STEM image of the precursors collected when the temperature was just raised to 160°C.

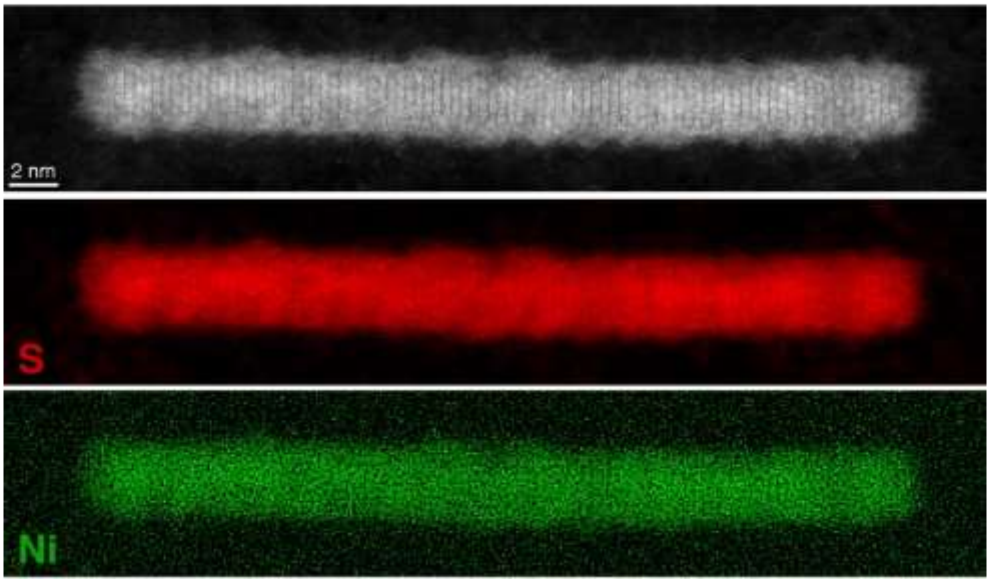

Fig. S4. ADF-STEM image and the corresponding electron energy loss spectroscopy (EELS) mapping images of NiS nanorod.

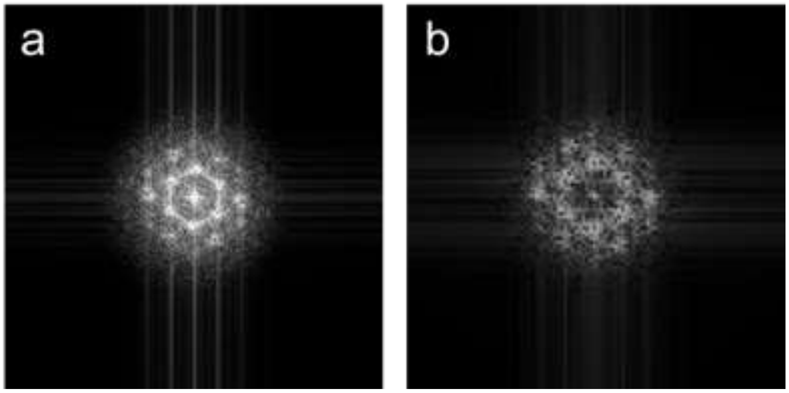

Fig. S5. (a) The corresponding FFT pattern of Fig. 1f, the thicker NiS nanorod in radial direction. (b) The corresponding FFT pattern of the inner part of the thicker NiS nanorod in radial direction.

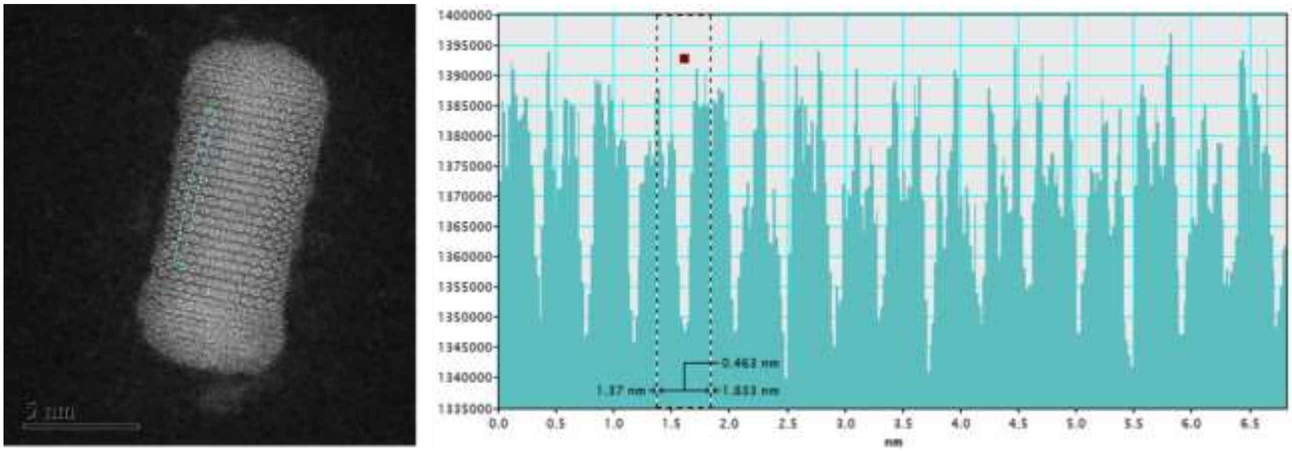

Fig. S6. The interlayer spacing of the lamellar structure form side view of the thicker NiS nanorod is measured to be ~0.46 nm.

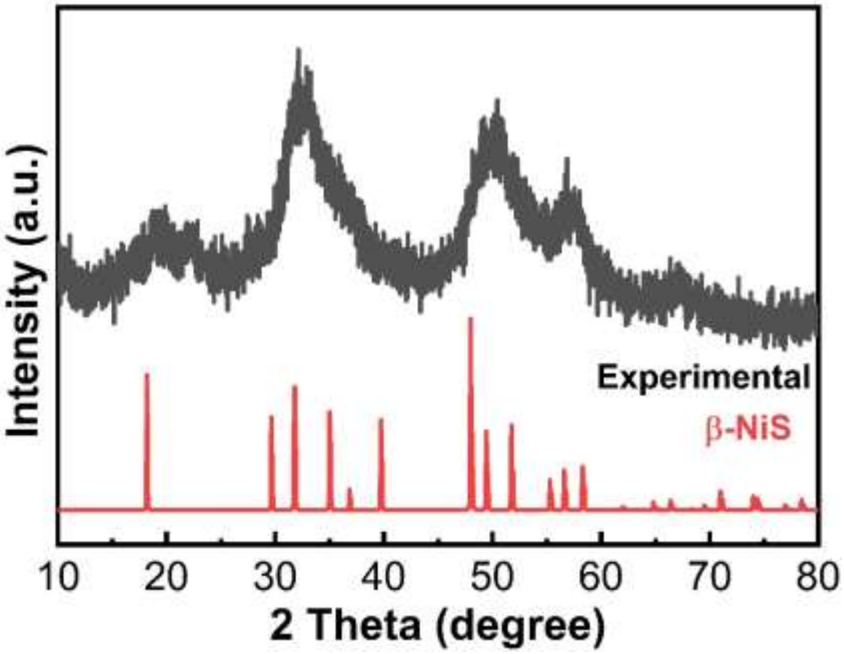

Supplementary Fig. 7. XRD patterns of NiS ultrafine nanorods, compared with the simulated pentacoordinate-NiS.

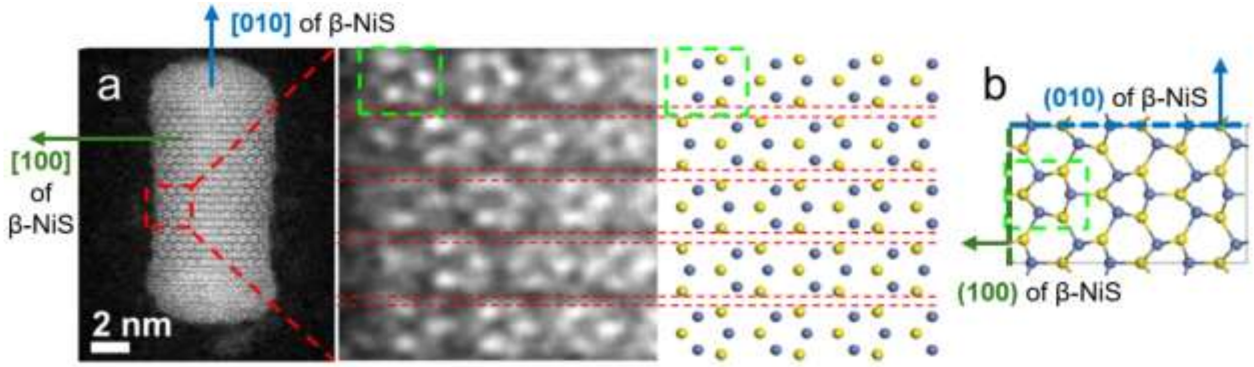

Supplementary Fig. 8. (a) STEM images and the symbolized Ni and S atoms. (b) The atomic structure of pentacoordinate β-NiS is displayed, showing both the [100] and [010] surface orientations. The blue and yellow spheres in a and b represent the Ni and S atoms, respectively.

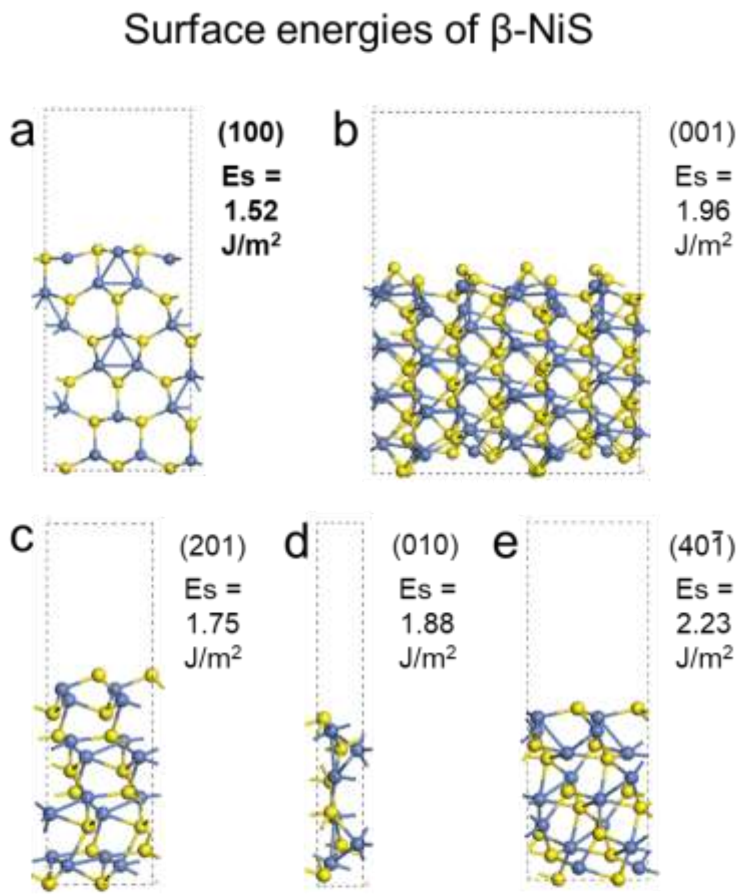

Supplementary Fig. 9. Illustration of differential surface energies in β-NiS. Blue and yellow spheres correspond to Ni and S atoms, respectively.

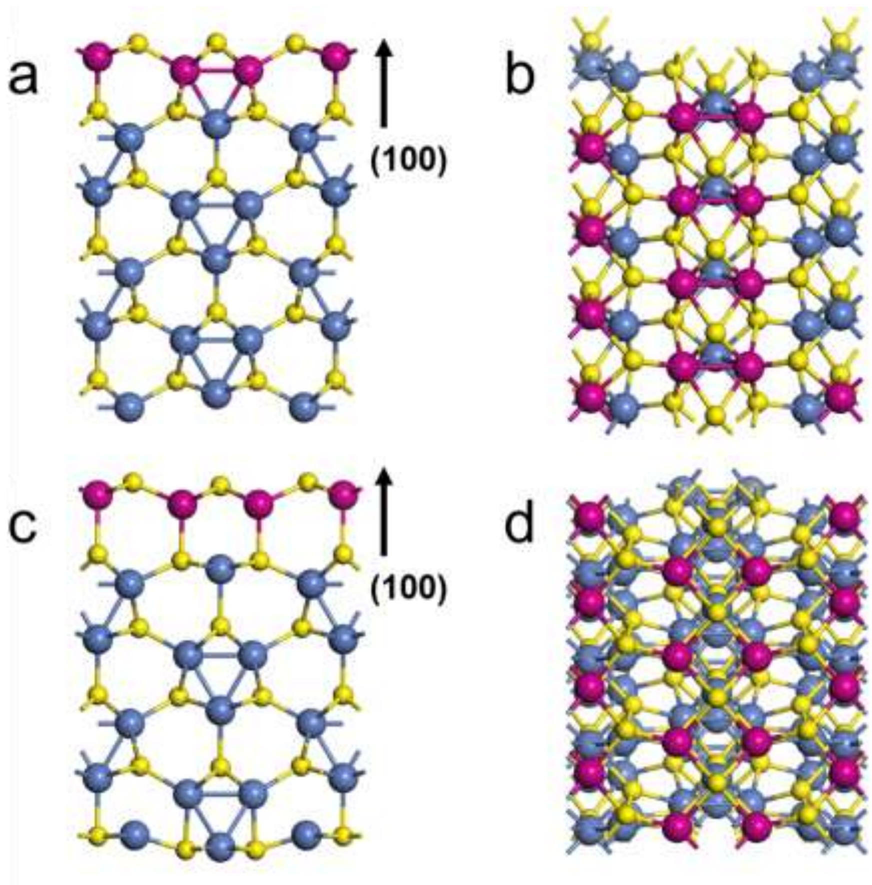

Supplementary Fig. 10. Depiction of the reconstruction process of the (100) surface slab of β-NiS. The spheres in blue and yellow signify Ni and S atoms, respectively, while the pink spheres represent the reconfigured Ni atoms at the surface.

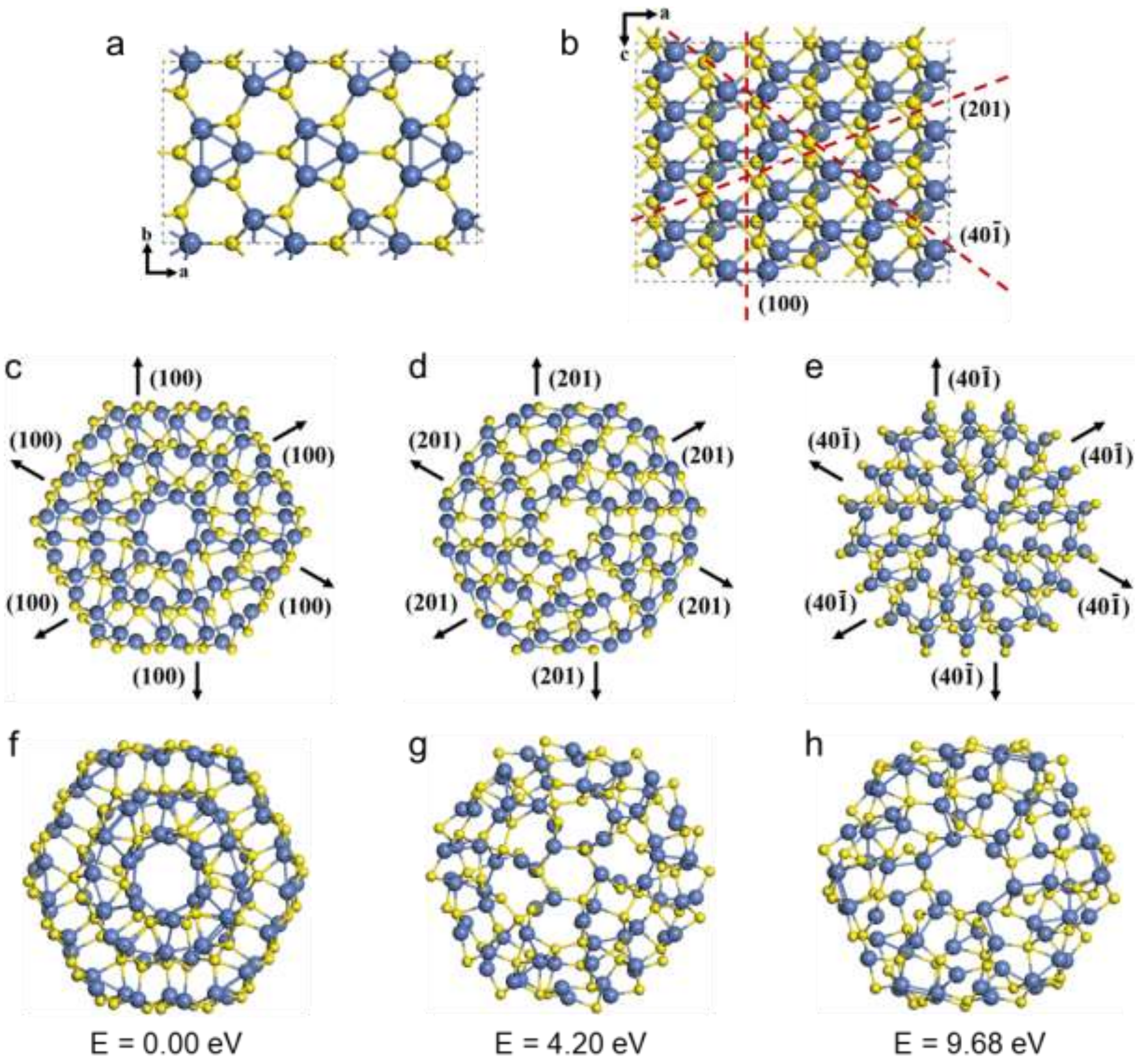

Supplementary Fig. 11. Comparative energy profiles of NiS nanorods with three potential surface exposures. (a, b) Display the NiS nanorod in (a) axial and (b) radial directions. (c-h) Illustrate atomic configurations of NiS nanorods with various surface exposures, specifically (c-e) pre-structural optimization and (f-h) post-structural optimization. Blue and yellow spheres symbolize Ni and S atoms, respectively.

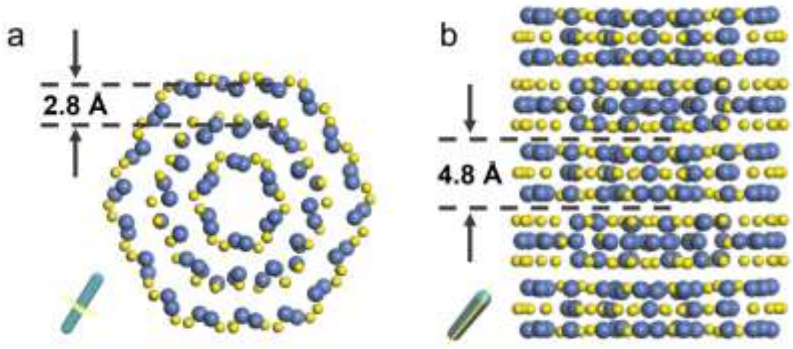

Supplementary Fig. 12. Computationally derived structures for NiS Nanorods in (a) radial and (b) axial Orientations. In the optimized structure, stripe spacing in the axial direction and ring spacing in the radial direction measure 4.8 Å and 2.8 Å, respectively.

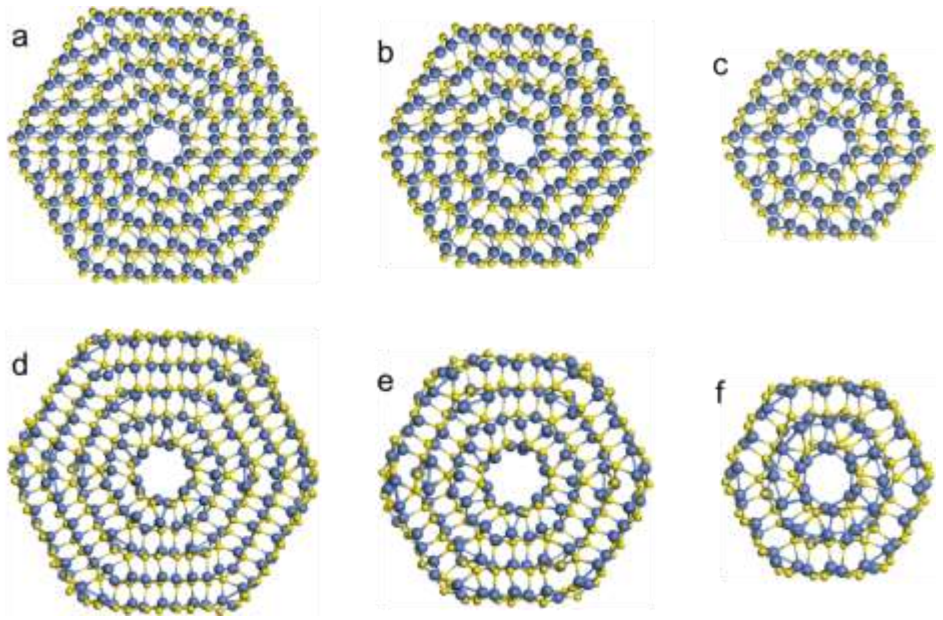

Supplementary Fig. 13. Atomic configurations of NiS nanorods with varying sizes (a-c) before and (d-f) after structural optimization. The blue and yellow balls represent the Ni and S atoms, respectively.

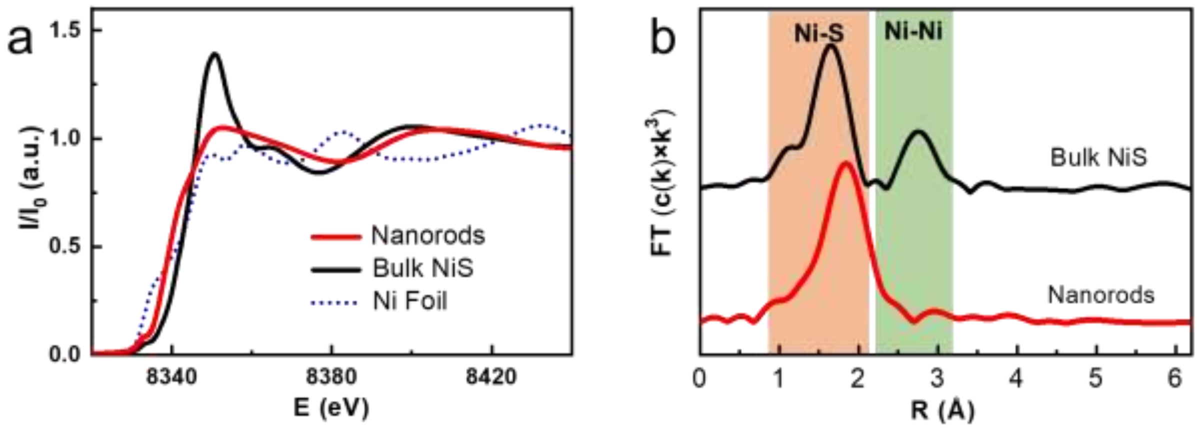

Supplementary Fig. 14. (a) Normalized XANES spectra of as-prepared NiS nanorod and the referential samples, showing the Ni K-edge. (b) k_3-weighted R-space Fourier transformed spectra from EXAFS.

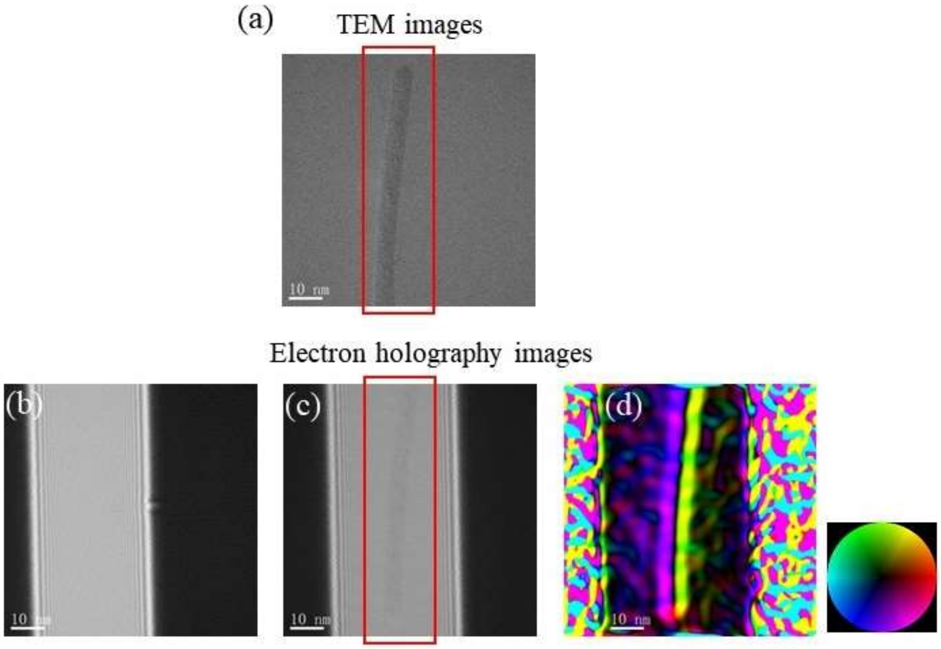

Supplementary Fig. 15. The electron holography image of the sample. (a) The TEM image of the sample. (b) The electron holography image of the vacuum region and (c) the overlapping region. (d) The exfoliated magnetism signal with the magnetic moment orientation represented by the color.

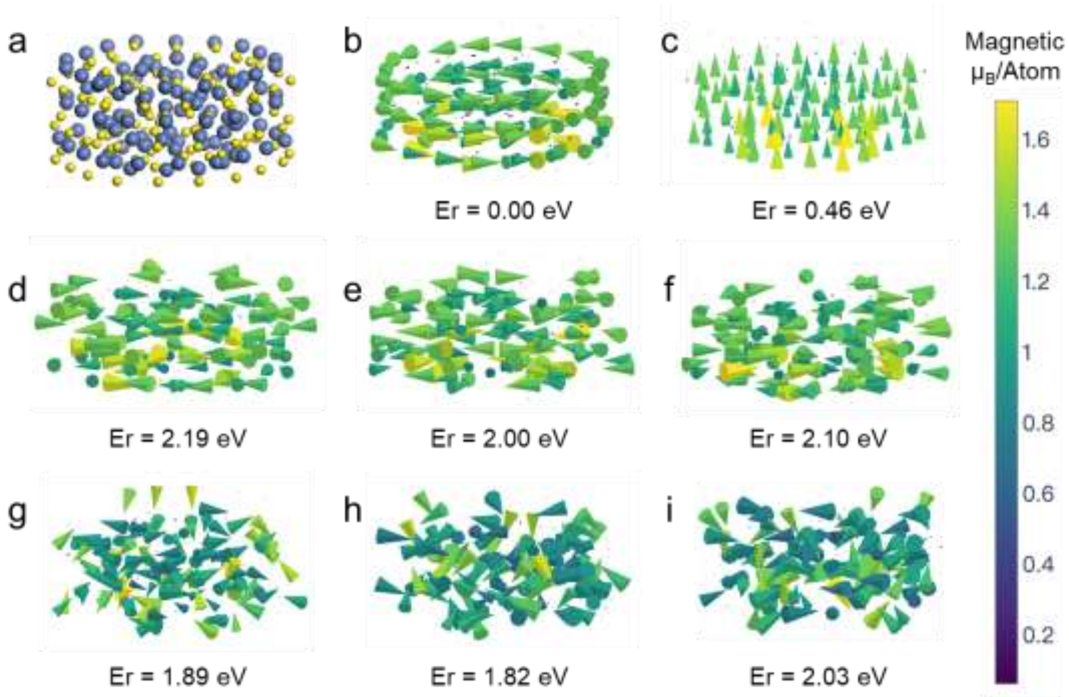

Supplementary Fig. 16. Energy comparisons across various magnetic configurations in noncollinear magnetism simulation. (a) Portrays the atomic configurations of NiS nanorods. (b) Stripe-Vortex mode. (c) Parallel mode. (d-f) The magnetic direction is random in the xy plane. (g-i) The magnetic direction is random in three-dimensional space. The blue and yellow balls represent the Ni and S atoms, respectively. The size and direction of the cones symbolize the magnitude and orientation of magnetism, respectively.

Supplementary Table S1. EXAFS fitting parameters at the Ni K-edge for various samples (S_0^2=0.76)

	Shell	CN	R (Å)	σ^2	ΔE_0 (eV)	R factor
**NiS Reference**	**Ni-S5**	**5**	**2.23±0.08**	**0.0081**	**-8.1±2.4**	**0.0113**
**Sample**	**Ni-S2**	**2.1±0.4**	**2.26±0.02**	**0.0018**	**-8.8±4.2**	**0.0139**
	**Ni-S3**	**3.2±0.5**	**2.17±0.02**	**0.0036**	**-1.1±3.3**	

CN: coordination numbers; *R*: interatomic distance; σ^2: Debye-Waller factors; ΔE_0: the inner potential correction. *R* factor: goodness of fit. S_0^2 was set to 0.76, according to the fitting of NiS reference by fixing CN as the known crystallographic value.

The acquired EXAFS data were normalized using the ATHENA module implemented in the IFEFFIT software packages. The k^3-weighted $\chi(k)$ data of Ni K-edge were Fourier transformed to real (R) space using a hanning windows (Δk = 1.0 $Å^{-1}$) to separate the EXAFS contributions from different coordination shells. The ARTEMIS code was used to obtain the quantitative structural parameters by least-squares fitting.
